# Testing lupus anticoagulants in a real-life scenario - a retrospective cohort study

**DOI:** 10.11613/BM.2017.030705

**Published:** 2017-08-28

**Authors:** Franz Ratzinger, Tanja Panic, Helmuth Haslacher, Thomas Perkmann, Klaus G. Schmetterer, Sabine Belik, Georg Maenner, Ingrid Pabinger, Peter Quehenberger

**Affiliations:** 1 Department of Laboratory Medicine, Medical University of Vienna, Vienna, Austria; 2 Clinical Division of Hematology and Hemostaseology, Department of Medicine I, Comprehensive Cancer Center Vienna, Medical University of Vienna, Vienna, Austria

**Keywords:** blood coagulation, blood coagulation tests, lupus coagulation inhibitor, partial thromboplastin time

## Abstract

**Introduction:**

Lupus anticoagulant (LAC) testing is challenging. Most data are derived from a well-controlled study environment with potential alterations to daily routines. The aim of this retrospective cohort study was to assess the capacity of various LAC screening tests and derived mixing tests to predict a positive result in subsequent confirmation tests in a large cohort of patients.

**Materials and methods:**

In 5832 individuals, we retrospectively evaluated the accuracy of the aPTT-A, aPTT-LA_screen_, aPTT-FS and dRVVT_screen_ and of their derived mixing tests in detecting a positive confirmation test result within the same blood specimen. The group differences, degree of correlation and the predictive accuracy of LAC coagulation tests were analysed using the Mann-Whitney U test, the Spearman-rank-correlation and by area under the receiver operating characteristic curve (ROC-AUC) analysis. ROC-AUCs were compared with the Venkatraman´s permutation test.

**Results:**

The pre-test probability of patients with clinically suspected LAC was 36% in patients without factor deficiency or anticoagulation therapy. The aPTT-LA_screen_ showed the best diagnostic accuracy with a ROC-AUC of 0.84 (95% CI: 0.82 – 0.86). No clear advantage of the dRVVT-derived mixing test was detectable when compared to the dRVVT_screen_ (P = 0.829). Usage of the index of circulating anticoagulant (ICA) did not improve the diagnostic power of respective mixing tests.

**Conclusions:**

Among the parameters evaluated, aPTT-LA_screen_ and derived mixing test parameters were the most accurate tests. In our study cohort, neither other mixing test nor the ICA presented any further advantage in LAC diagnostics.

## Introduction

The detection of lupus anticoagulants (LAC) is based on interference testing of the coagulation cascade and therefore testing it proves challenging (*1*). Several guidelines and expert recommendations exist proposing LAC testing in a stepwise procedure including screening, mixing and confirmatory tests (*2*–*4*). Since no individual screening test presents with a high diagnostic accuracy, most guidelines recommend the performance of two tests for the initial screening, including a test based on the diluted Russell Viper venom time (dRVVT) and a LAC-sensitive activated partial thromboplastin time (aPTT-LA_screen_) containing low amounts of phospholipids. The aPTT-LA_screen_ appears to display higher sensitivity and the dRVVT, described as being the most robust assay for LAC testing, might possess a higher specificity for detecting LAC (*4*, *5*). As an in-house procedure, the performance of a mixing test is advocated with a 1:1 ratio between patient plasma (PP) and pooled normal plasma (PNP). With regard to the mixing test, there are some differences in the actual guidelines. While the International Society on Haemostasis and Thrombosis (ISTH) and the British Committee for Standards in Haematology (BCSH) recommend a screening-mixing-confirmation test order, the Clinical and Laboratory Standards Institute (CLSI) suggests the screening, confirmation and mixing test order (*2*, *4*, *6*). According to the latter guideline, the mixing test should be omitted in samples without evidence of other causes of elevated clotting times (CT). Generally, mixing tests are used to differentiate between coagulation factor deficiencies and coagulation inhibitors or treatment with heparin as the cause of a prolonged CT. However, due to dilution effects, a negative mixing test result does not rule out the presence of a “weak” LAC (*6*). Likewise, false-positive mixing tests can result from interference by therapeutic anticoagulants (*7*). Confirmation testing should be conducted with an increased concentration of phospholipids compared to screening tests, and a ratio between the CT with low and high concentrations of phospholipids should be calculated.

There is no agreement between the guidelines regarding LAC testing for patients taking vitamin K antagonists (VKA) or heparin. Generally, factor deficiency in patients on VKA with an international normalized ratio (INR) above 1.5 can affect LAC testing and the results have to be taken with caution. While CLSI and ISTH suggest caution with respect to the results of LAC testing of patients under heparin therapy, the BCSH recommends no LAC testing of such samples at all.

Due to high demands on laboratory facilities, the incurred costs and the wide availability of integrated confirmation testing, the need for mixing tests has to be carefully evaluated. However, there is hardly any data for a suspected LAC patient cohort with representative pre-test probability of having LAC. Hence we conducted a retrospective cohort study to evaluate the predictive capacity of various LAC screening tests and derived mixing tests in detecting a positive confirmation test result within the same blood sample in 5832 patients without any factor deficiency or anticoagulation therapy (dataset A), in patients with heparin therapy (dataset B) and in patients on VKA therapy (dataset C). Whether or not positive results of confirmation testing could be confirmed at a later time point (as a criterion for diagnosing antiphospholipid syndrome, APL) was not the goal of this assessment.

## Materials and methods

### Study design

This retrospective cohort study was performed between 2010 and 2014 at the Vienna General Hospital, Medical University of Vienna, Austria, a tertiary teaching hospital with 2145 beds. The study was approved by the local ethics committee (EC-Number: 1197/2013) and was conducted in accordance with the ethical principles of the Declaration of Helsinki.

### Subjects

Due to the retrospective design of the study, informed consent was not obtained from study participants. All consecutive patients older than 18 years of age with clinical suspicion of having LAC from whom the treating physician requested LAC testing were included. Before requesting coagulation testing, clinicians were obligated to document any anticoagulant therapy. Based on this information, INR measurements (for patients on VKA) or anti-Xa-measurements (for patients on heparin therapy) were performed. Patients without a complete LAC testing panel, patients with known coagulation interfering antibodies other than LAC or known haemophilia A, B or contact factor deficiency, patients under direct oral anticoagulants or heparin therapy with anti-Xa activities above 1 IU/mL were excluded. A complete LAC panel included the following assessment: prothrombin time (PT) according to Owren and Quick, thrombin clotting time (TCT), fibrinogen, LAC screening tests including aPTT-A (using STA–PTTA reagent), aPTT-FS (using Actin FS reagent), aPTT-LA_screen_ or dRVVT_screen_, and one derived mixing test as well as the aPTT-LA- and dRVVT- confirmation tests (see Supplementary figure 1). No patients were included more than once, as only the first LAC request for a patient was included during the study period.

**Figure 1:**
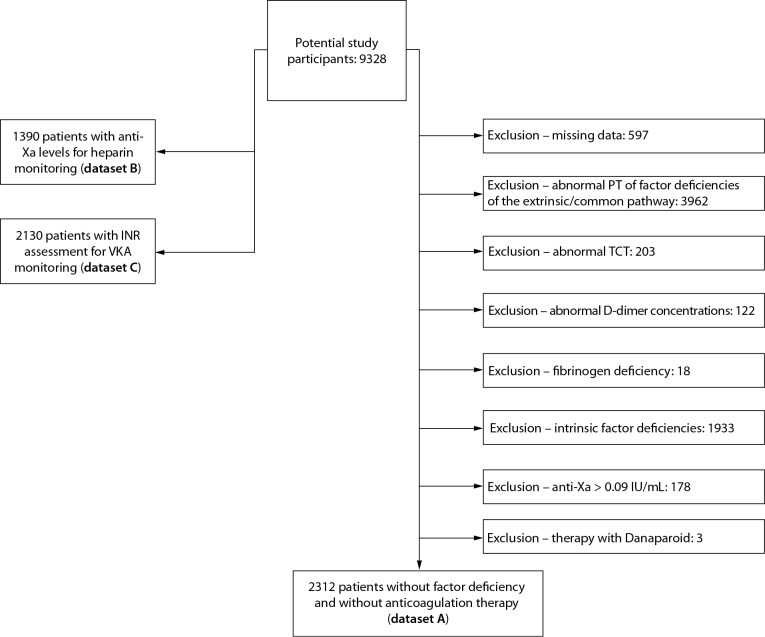
Study recruitment process. VKA - vitamin K antagonist. PT - prothrombin time. TCT - thrombin clotting time.

To increase the within-group homogeneity and to address the discrepancy between available literature and recommendations with the clinical daily practice, three datasets (Figure 1) were established including patients without factor deficiency and without evidence of anticoagulation therapy (dataset A), patients with anti-Xa assessment for heparin therapy monitoring (dataset B) and patients with INR assessment for the monitoring of VKA therapy (dataset C).

Between January 2010 and December 2014, LAC testing was conducted in 9328 patients. In 597 patients, no full LAC testing panel was available. In further 178 patients, the anti-Xa activity for monitoring heparin therapy was above 1 IU/mL (Figure 1). For dataset A, patients with abnormal PT or extrinsic factor deficiencies (N = 3962), intrinsic factor deficiencies (N = 1933), abnormal TCT (N = 203), abnormal D-dimer concentrations (N = 122), fibrinogen deficiency (N = 18) and patients with Danaparoid therapy (heparinoid, inhibiting activated factor X, N = 3) were excluded. Among the patients of dataset A (N = 2312, female-to-male ratio: 52% to 48%), 35.8% presented with post-test evidence of LAC (N = 828, Table 1, dataset A). Among the total study population, LAC testing and anti-Xa activities for monitoring heparin therapy were evaluated in 1390 patients (dataset B, female-to-male ratio: 53% to 47%). Further 2130 patients on VKA therapy were included (dataset C). In dataset C, LAC positivity rate was 13% and the female-to-male ratio was 49% to 51%. We pre-defined INR values as sub-therapeutic (INR = 1.31 - 1.99; N = 536), therapeutic (INR = 2.00 - 3.50; N = 1324) and supra-therapeutic (INR > 3.51; N = 270). The comparison of the dRVVT was not performed, since the dRVVT was routinely not assessed in patients with (acquired) FX deficiency.

**Table 1:** Coagulation parameters assessed in dataset A

	**Parameter**	**LAC negative** **N = 1484**	**LAC positive** **N = 828**	P-value*	**ROC** **(95% CI)**
**All patients**	PT Owren (%)	103.0 (91.0 – 117.2)	106.5 (95.0 – 123.0)	< 0.001	0.56 (0.53 – 0.58)
	PT Quick (%)	91.0 (85.0 – 98.0)	92.0 (85.0 – 99.0)	0.363	0.51 (0.49 – 0.54)
	TCT (s)	13.7 (13.1 – 14.5)	13.7 (13.0 – 14.4)	0.053	0.48 (0.45 – 0.50)
	aPTT-A (s)	43.5 (41.0 – 46.2)	48.8 (44.4 – 56.5)	< 0.001	0.76 (0.74 – 0.78)
	aPTT-LA (s)	49.9 (45.8 – 54.4)	61.2 (54.8 – 72.3)	< 0.001	0.84 (0.82 – 0.86)
	aPTT-FS (s)	39.6 (36.7 – 41.9)	37.0 (34.2 – 40.3)	< 0.001	0.65 (0.62 – 0.67)
	dRVVT (s)	45.8 (41.3 – 50.6)	56.9 (50.3 – 65.8)	< 0.001	0.81 (0.79 – 0.83)
	Fibrinogen (g/L)	3.77 (3.08 – 4.61)	4.27 (3.47 – 5.50)	< 0.001	0.62 (0.59 – 0.64)
	FVIII (%)	137 (102 – 190)	174 (132 – 234)	< 0.001	0.65 (0.63 – 0.67)
	FIX (%)	109 (91 – 129)	123 (102 – 147)	< 0.001	0.61 (0.59 – 0.64)
	FXI (%)	93 (80 – 107)	98 (84 – 115)	< 0.001	0.57 (0.54 – 0.59)
	FXII (%)	92 (77 – 113)	96 (80 – 119)	0.003	0.54 (0.51 – 0.56)
**Patients grouped according to the mixing test applied**					
**aPTT-A**		**N = 512**	**N = 110**		
	aPTT-A (s)	45.0 (43.4 – 47.6)	50.8 (47.0 – 57.4)	< 0.001	0.78 (0.73 – 0.83)
	∆Mix–PNP (s)	4.3 (2.6 – 6.2)	8.2 (5.5 – 11.1)	< 0.001	0.79 (0.75 – 0.84)
	ICA (%)	9.3 (5.8 – 12.9)	16.1 (11.3 – 20.8)	< 0.001	0.77 (0.72 – 0.82)
**aPTT-LA**		**N = 375**	**N = 476**		
	APTT-LA_screen_ (s)	56.4 (53.4 – 60.3)	67.5 (59.3 – 81.0)	< 0.001	0.80 (0.77 – 0.83)
	∆Mix–PNP(s)	6.7 (4.7 – 9.6)	15.8 (10.3 – 25.3)	< 0.001	0.84 (0.82 – 0.87)
	ICA (%)	11.9 (8.3 – 16.2)	23.2 (16.4 – 33.6)	< 0.001	0.83 (0.80 – 0.85)
**dRVVT**		**N = 118**	**N = 230**		
	dRVVTscreen (s)	58.7 (56.2 – 62.0)	66.8 (60.3 – 76.6)	< 0.001	0.77 (0.72 – 0.82)
	∆Mix–Norm (s)	5.0 (2.9 – 7.0)	9.1 (5.8 – 15.8)	< 0.001	0.77 (0.71 – 0.82)
	ICA (%)	8.2 (4.9 – 11.7)	13.3 (9.2 – 21.1)	< 0.001	0.74 (0.69 – 0.80)
**aPTT-FS^†^**		**N = 479**	**N = 12**		
	aPTT-FS (s)	42.0 (40.5 – 43.8)	44.5 (42.7 – 47.4)	0.002	0.77 (0.65 – 0.88)
	∆Mix–Norm (s)	1.5 (0.4 – 2.7)	1.8 (0.9 – 4.3)	0.162	0.62 (0.46 – 0.80)
	ICA (%)	3.5 (1.0 – 6.3)	4.3 (2.0 – 8.7)	0.223	0.60 (0.44 – 0.77)
Data are derived from patients without factor deficiency or anticoagulant therapy. P < 0.05 considered statistically significant (after application of the Bonferroni-Holm correction). *Comparison between patients with and without lupus anticoagulants (LAC), using Mann–Whitney U test. ROC - area under the receiver operating characteristics curve, presented with 95% confidence intervals (CI). PT Owren - prothrombin time according to Owren. PT Quick - prothrombin time according to Quick. TCT - thrombin clotting time. aPTT-A - activated partial thromboplastin time determined using STA–PTTA reagent (Roche Diagnostics). aPTT-FS - activated partial thromboplastin time determined using Actin FS (Siemens Healthcare GmbH). aPTT-LA - LAC-sensitive activated partial thromboplastin time. dRVVT - diluted Russell Viper venom time. PNP - pooled normal plasma. ICA - index of circulating anticoagulant. ^†^aPTT-FS is not recommended for LAC-testing.					

### Methods

Blood samples were collected in siliconized tubes (Vacuette®, Greiner Bio-One, Kremsmünster, Austria) with 0.023 M pre-filled sodium citrate (final concentration). In accordance to the H-60-A CLSI guideline, all samples were first centrifuged at 2500xg for 15 minutes at 15 °C (Mikro 220 R, Bartelt GmbH, Graz, Austria), transferred to a secondary polypropylene tube (5 mL, 75 x 13mm PP, Sarstedt AG & Co, Nümbrecht, Germany) and again centrifuged under the same conditions to obtain platelet-poor plasma (platelet counts: < 10 x10^9^/L). Except LAC confirmatory testing, all coagulation analyses were conducted by using freshly drawn samples within 4 hours after blood taking. Thawed plasma specimens, stored below - 70°C upon analysis, were used for LAC confirmatory testing. Reagents, analysers and established reference ranges are summarized in Supplementary table 1 and quality control materials as well as their coefficient of variation are presented in Supplementary table 2. All test procedures were conducted according to the manufacturer´s recommendations. Using a coagulometric method, PT Owren (Normotest, Technoclone GmbH, Vienna, Austria), PT Quick (Thromborel S, Siemens Healthcare GmbH, Erlangen, Germany), TCT (STA-Thrombin, Roche Diagnostics, Rotkreuz, Switzerland), fibrinogen (STA–liquid Fib, Roche Diagnostics), activated partial thromboplastin time (aPTT-A, STA–PTTA, Roche Diagnostics), aPTT-LA_screen_ (PTT LA, Roche Diagnostics), factor sensitive activated partial thromboplastin time (aPTT-FS, ACTIN FS, Siemens Healthcare GmbH, Erlangen, Germany), dRVVT (DRVV SCREEN 2 reagent, Life Diagnostics, West Chester, USA) and dRVVT confirmation test (dRVVT_confirm_, DRVV CONFIRM reagent, Life Diagnostics, West Chester, USA) were assessed on the STA-R Evolution (Diagnostica Stago S.A.S, Asnières sur Seine, France). The aPTT-LA confirmation test (LA_confirm_, Staclot LA, Roche Diagnostics) was applied on the MC10 PLUS (ABW Medizin und Technik GmbH, Lemgo, Germany).

On the Sysmex CA-7000 (Siemens Healthcare GmbH, Erlangen, Germany) analyser, the coagulation factors VIII (FVIII), IX (FIX), XI (FXI) and XII (FXII) were determined by one-stage coagulometric methods in a multidilution procedure using Actin FS reagent (Siemens Healthcare GmbH, Erlangen, Germany) and appropriate factor deficient plasmas (for FVIII and FIX: Technoclone GmbH, and for FXI and FXII: Siemens Healthcare GmbH). For the LAC screening procedure the aPTT-A, aPTT-FS, aPTT-LA_screen_, and dRVVT_screen_ were applied. The LAC screening parameter with the largest absolute difference between our established upper cut-off value and the measured CT was used for the mixing test procedure. The results of the mixing tests are presented in Table 1, Supplementary table 3 and Supplementary table 4 as subgroups of the corresponding total population. In dataset A, the mixing test was performed using the aPTT-A in 622 patients, the aPTT-LA in 851 patients, the aPTT-FS in 491 patients and the dRVVT in 348 patients (Table 1). The same approach was used in dataset B (see Supplementary table 3) and dataset C (see Supplementary table 4). Due to the low number of LAC-positives in subgroups of datasets B and C, the accuracy of the aPTT-FS derived mixing test parameters was not assessed. According to the CLSI H60-AE guideline, normal pool plasma (PNP) prepared from plasma samples of 20 apparently healthy volunteers was used for mixing test studies. The mixing test was performed by mixing one part of patient plasma with one part of PNP using a 1:1 ratio on a STA-R Evolution (Diagnostica Stago S.A.S, Asnières sur Seine, France). The difference between PNP and the corresponding 1:1 mixing sample (∆Mix–PNP) was computed. Further the index of circulating anticoagulant (ICA, Rosner Index) was calculated using the following formula (*8*):


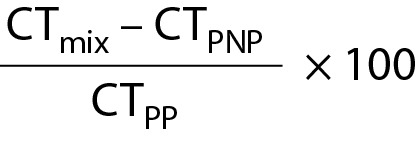


For the LA_confirm_ (Staclot LA reagent, Roche Diagnostics, Rotkreuz, Switzerland) assessment, patient plasma is mixed with reagent buffer 1 (tube 1) or with reagent buffer 2 (containing phospholipids, tube 2) and incubated for 9 minutes at 37 °C. Afterwards human plasma (reagent 3) was added to both tubes, and incubated for one minute and finally lyophilized PTT-LS reagent (reagent 4) was added and incubated for another five minutes. CTs were measured after adding CaCl_2_ (0.025 M, pre-incubated at 37 °C). The dRVVT_confirm_ (DRVV Confirm reagent, Life Diagnostics, West Chester, USA) assessment was again carried out in two separate reactions. Patient plasma was mixed either with DRVV-Screen reagent or with the DRVV-Confirm reagent containing excess phospholipid concentration (all pre-incubated at 37 °C). After adding the pre-incubated starting reagent, both CTs were measured and the ratio between CT_screen_ and CT_confirm_ was calculated.

Both confirmation tests, which are widely used, were applied in all patients. Confirmation testing and occurrence of LAC was considered positive when either LA_confirm_ decreased CT (CT_tube1_ – CT_tube2_) for more than 3 s or the ratio of dRVVT_screen_ to the dRVVT_confirm_ was above 1.25 within the same sample. The LAC confirmatory cut-offs were established by in-house evaluations. According to our standard operation procedures, the dRVVT_screen_ was not assessed in patients with hereditary or acquired FX deficiency. All analyses were performed under standardized conditions at the Department of Laboratory Medicine, which maintains a certified (according to ISO 9001:2008) and accredited (according to ISO 15189:2008) quality management system.

### Statistical analysis

**Figure 2:**
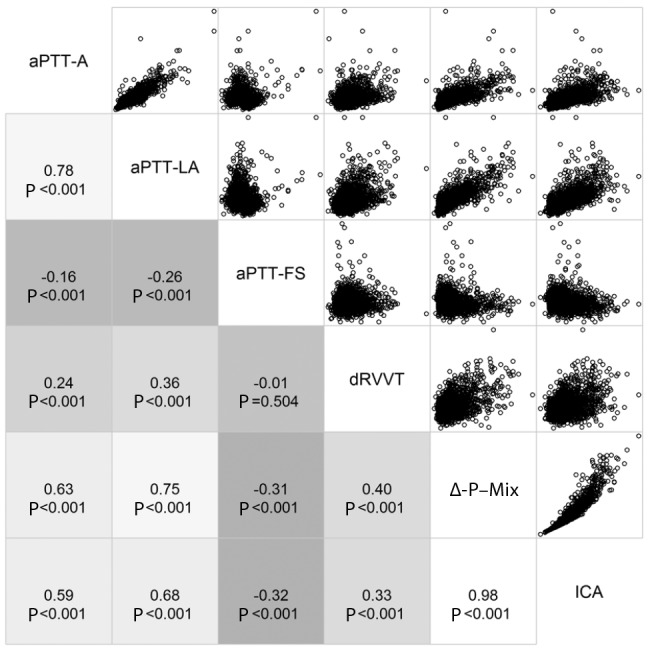
Correlation of LAC parameters.  Spearman correlation coefficients ρ are presented with their corresponding significance levels; for a better overview of the scatterplots, 1% outliers were eliminated according to the Mahalanobis distance criterion using the multivariate outlier package; aPTT-A, aPTT-LA, aPTT-FS and dRVVT are the screening tests using all patients presented in Table 1.

Metric data are given as median (interquartile range, IQR) and occurrence of normal distribution was analysed using the Shapiro-Wilk test. Group-differences of metric data were compared by applying Mann-Whitney U tests. The diagnostic performance of parameters was analysed using the area under the receiver operating characteristics curve (ROC-AUC). Since no perfect LAC screening test exists and the majority of relevant guidelines recommend the utilization of at least two screening tests, the correlation between LAC screening parameters was assessed by the Spearman correlation coefficients and graphically presented as a correlogram (Corrgram package, see Figure 2). To enable a better overview of scatterplots, 1% outliers were eliminated according to the Mahalanobis distance criterion using the multivariate outlier package. For finding an optimal cut-off point of a diagnostic test, the Youden Index method was used. 95% confidence intervals (CI) of the ROC-AUC and of binary outcome measures were computed with 2000 stratified bootstrap-replicates (*9*). Dependent or independent ROC-AUCs were compared using a distribution-free permutation test according to Venkatraman, implemented in the pROC package. Statistical significance was defined as P-values less than 0.05. Where appropriate, an error related to multiple testing was corrected using the Bonferroni-Holm method. All statistical analysis was conducted using R (Version 3.2.2, R Foundation for Statistical Computing, Vienna, Austria).

## Results

### LAC testing in patients without factor deficiency of anticoagulation therapy

**Figure 3:**
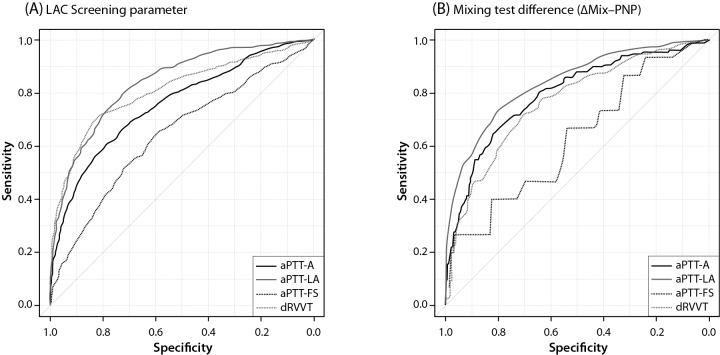
Comparison of ROC-AUCs of LAC screening parameters.  (A) Dependent ROC-AUCs with corresponding 95% confidence intervals (all dataset A patients); aPTT-A: 0.76 (0.74 – 0.78), aPTT-LA 0.84 (0.82 – 0.86), aPTT-FS: 0.65 (0.62 – 0.67), dRVVT: 0.81 (0.79 – 0.83).  (B) Independent ROC-AUCs with corresponding 95% confidence intervals (patient subgroups according to the mixing test performed): aPTT-A: 0.79 (0.75 – 0.84), aPTT-LA: 0.84 (0.82 – 0.87), aPTT-FS: 0.62 (0.46 – 0.79), dRVVT: 0.77 (0.71 – 0.82). aPTT-FS is not recommended for LAC-testing.

In dataset A, including 2312 patients without coagulation factor deficiency and without evidence of anticoagulation therapy, the LAC confirmation test positivity was 35.8%. The highest ROC-AUCs among the screening parameter revealed the aPTT-LA_screen_ with 0.84 (95% CI: 0.82 – 0.86, see Table 1, Figure 3), which was significantly better than the dRVVT_screen_ with 0.81 (95% CI: 0.79 – 0.83, P < 0.001, see Table 2). Among the screening parameters, the aPTT-A and aPTT-LA_screen_ presented a high correlation to each other (ρ = 0.78, P < 0.001; Figure 2).

**Table 2:** Comparison of the predictive accuracy of LAC screening and mixing test derived parameters

	**Parameter**		**aPTT-A**		**aPTT-LA_screen_**		**dRVVT_screen_**
**All patients**	**aPTT-A**		ROC-AUC: 0.76 (0.74 – 0.78)		P < 0.001		P < 0.001
	**aPTT-LA_screen_**		P < 0.001		ROC-AUC: 0.84 (0.82 – 0.86)		P = 0.037
	**dRVVT_screen_**		P < 0.001		P = 0.037		ROC-AUC: 0.81 (0.79 – 0.83)
**Patients grouped according to the mixing test applied**							
**aPTT-A**		**aPTT-A**		**∆Mix–PNP**		**ICA**	
	**aPTT-A**	ROC-AUC: 0.78 (0.73 – 0.83)		P = 0.816		P = 0.384	
	**∆Mix–PNP(s)**	P = 0.816		ROC-AUC: 0.79 (0.75 – 0.84)		P < 0.001	
	**ICA**	P = 0.384		P < 0.001		ROC-AUC: 0.77 (0.72 – 0.82)	
**aPTT-LA**		**APTT-LA_screen_**		**∆Mix–PNP**		**ICA**	
	**APTT-LA_screen_**	ROC-AUC: 0.80 (0.77 – 0.83)		P = 0.005		P = 0.257	
	**∆Mix–PNP**	P = 0.005		ROC-AUC: 0.84 (0.82 – 0.87)		P < 0.001	
	**ICA**	P = 0.257		P < 0.001		ROC-AUC: 0.83 (0.80 – 0.85)	
**dRVVT**		**dRVVT_screen_**		**∆Mix–PNP**		**ICA**	
	**dRVVT_screen_**	ROC-AUC: 0.77 (0.72 – 0.82)		P = 0.829		P = 0.247	
	**∆Mix–PNP**	P = 0.829		ROC-AUC: 0.77 (0.71 – 0.82)		P < 0.001	
	**ICA**	P = 0.247		P < 0.001		ROC-AUC: 0.74 (0.69 – 0.80)	
ROC-AUC - area under the receiver operating characteristics curve, presented with 95% confidence intervals (CI), of the screening test and mixing test parameters (dataset A patients). P-values were calculated using the permutation test according to Venkatraman for dependent ROC-AUCs. P < 0.05 was considered statistically significant (after applying the Bonferroni-Holm correction). aPTT-A - activated partial thromboplastin time determined using STA–PTTA reagent (Roche Diagnostics). aPTT-LA - LAC-sensitive activated partial thromboplastin time. dRVVT - diluted Russell Viper venom time. PNP - pooled normal plasma. ICA - index of circulating anticoagulant. aPTT-FS is not displayed since this test in not recommended for LAC testing.							

The mixing test was performed only in the test system showing the largest absolute deviance from the upper reference range, which are presented as subgroups in Table 1 and Table 2. Within the mixing test, the best ROC-AUC was found in the aPTT-LA ∆Mix–PNP with 0.84 (95% CI: 0.82 – 0.87; Figure 3), which was significantly better than the ROC-AUC of the dRVVT ∆Mix–PNP (0.77, 95% CI: 0.71 – 0.82, P = 0.016). Furthermore, the aPTT-LA ∆Mix–PNP and aPTT-A ∆Mix–PNP with 0.79 ROC-AUC (95% CI: 0.75 – 0.84) had significantly better diagnostic accuracy than the aPTT-FS ∆Mix–PNP (0.62, 95% CI: 0.46 – 0.79, P = 0.002 and P = 0.019, respectively), which is not recommended for LAC testing. When comparing the LAC screening test with the corresponding ∆Mix–PNP mixing test result, significant improvement was only found for the aPTT-LA (P = 0.005, see Table 2), while the ∆Mix–PNP did not improve diagnostic accuracy in the aPTT-A (P = 0.816), and the dRVVT_screen_ (P = 0.829). Calculation of ICA significantly decreased the performance of the ∆Mix–PNP parameters in the aPTT-A, aPTT-LA, and dRVVT (all: P < 0.001). Table 3 presents an overview of the diagnostic performance of binarized mixing test results. At an optimal cut-off threshold, the aPTT-LA ∆Mix–PNP (cut-off: 10.70 s) presented with 73.5% sensitivity and 82.4% specificity, while the dRVVT resulted in 67.8% sensitivity and 76.3% specificity (cut-off: 6.75 s). A trend analysis of resulting sensitivities and specificities over the course of 12 seconds is shown in Table 4.

**Table 3:** Diagnostic accuracy of the mixing tests (∆Mix–PNP) assessed

	**APTT-A** **N = 622**	**APTT-LA** **N = 851**	**dRVVT** **N = 348**	**APTT-FS^†^** **N = 491**
**ROC-AUC (95% CI)**	0.79 (0.75 – 0.84)	0.84 (0.82 – 0.87)	0.77 (0.71 – 0.82)	0.62 (0.45 – 0.79)
Cut-off (s) *	6.35 (4.65 – 7.95)	10.70 (9.65 – 11.85)	6.75 (5.90 – 10.15)	1.75 (0.45 – 6.45)
**Sensitivity (%)**	70.9 (54.6 – 88.2)	73.5 (67.2 – 78.8)	67.8 (45.2 – 78.7)	66.7 (16.7–100.0)
**Specificity (%)**	78.4 (57.4 – 90.8)	82.4 (76.5 – 88.0)	76.3 (62.7 – 94.1)	60.4 (24.8 – 99.8)
**NPV (%)**	92.6 (90.0 – 96.0)	71.0 (67.2 – 74.8)	54.6 (46.3 – 62.4)	98.8 (97.9 – 100.0)
**PPV (%)**	40.9 (30.7 – 58.0)	84.2 (80.5 – 88.1)	85.0 (79.7 – 94.3)	5.0 (3.0 – 75.0)
ROC-AUC - area under the receiver operating characteristics curve, presented with 95% confidence intervals (95% CI). NPV - negative predictive value, PPV - positive predictive value. aPTT-A - activated partial thromboplastin time determined using STA–PTTA reagent (Roche Diagnostics). aPTT-LA - LAC-sensitive activated partial thromboplastin time. dRVVT - diluted Russell Viper venom time. aPTT-FS - activated partial thromboplastin time determined using Actin FS (Siemens Healthcare GmbH). aPTT-A *vs*. aPTT-LA: P = 0.095, aPTT-A *vs*. aPTT-FS: P = 0.019, aPTT-A *vs*. dRVVT: P = 0.635, aPTT-LA *vs*. aPTT-FS: P = 0.002, aPTT-LA *vs*. dRVVT: P = 0.016, aPTT-FS *vs*. dRVVT: P = 0.052. *Cut-off value assessed via the Youden index method; ^†^aPTT-FS is not recommended for LAC-testing.				

**Table 4:** Trend analysis of the diagnostic sensitivity and specificity of ∆Mix–PNP

**Threshold (s)**	**Sensitivity (95% CI)**	**Specificity (95% CI)**	**Sensitivity (95% CI)**	**Specificity (95% CI)**
**aPTT-A**		**aPTT-LA**	
1	98.2 (95.5 – 100.0)	8.4 (6.1 – 10.9)	99.4 (98.5 – 100.0)	5.1 (2.9 – 7.2)
**2**	94.6 (90.0 – 98.2)	15.6 (12.5 – 18.8)	99.2 (98.3 – 99.8)	10.9 (7.7 – 14.1)
**3**	94.6 (90.0 – 98.2)	29.3 (25.6 – 33.4)	97.7 (96.2 – 99.0)	16.5 (12.8 – 20.3)
**4**	89.1 (82.7 – 94.6)	45.3 (41.0 – 49.2)	96.9 (95.2 – 98.3)	21.1 (17.1 – 25.3)
**5**	80.9 (73.6 – 88.2)	60.4 (56.3 – 64.5)	95.8 (93.9 – 97.5)	27.5 (23.2 – 32.3)
**6**	70.0 (60.9 – 79.1)*	73.6 (69.5 – 77.3)*	92.7 (90.1 – 95.0)	43.2 (38.7 – 48.3)
**7**	60.0 (50.9 – 69.1)*	83.6 (80.3 – 86.9)*	88.5 (85.5 – 91.2)	52.3 (47.2 – 57.3)
**8**	54.6 (45.5 – 63.6)	89.7 (87.1 – 92.4)	84.7 (81.3 – 87.8)	62.4 (57.3 – 67.2)
**9**	40.0 (30.9 – 49.1)	93.0 (90.8 – 95.1)	79.8 (75.8 – 83.4)	71.7 (66.9 – 76.3)
**10**	32.7 (23.6 – 41.8)	95.1 (93.2 – 96.9)	76.1 (72.1 – 79.8)*	77.6 (73.3 – 81.6)*
**11**	26.4 (18.2 – 34.6)	96.1 (94.3 – 97.7)	72.1 (68.1 – 76.1)*	82.7 (78.7 – 86.4)*
**12**	23.6 (15.5 – 31.8)	97.1 (95.5 – 98.4)	65.8 (61.6 – 70.0)	86.1 (82.7 – 89.3)
	**dRVVT**		**aPTT-FS^†^**	
**1**	97.8 (95.7 – 99.6)	11.9 (5.9 – 18.6)	66.7 (41.7 – 91.7)*	37.0 (32.4 – 41.1)*
**2**	95.7 (92.6 – 98.3)	18.6 (11.9 – 25.4)	41.7 (16.7 – 66.7)*	61.2 (56.8 – 65.6)*
**3**	93.0 (89.6 – 96.1)	25.4 (18.6 – 33.9)	33.3 (8.3 – 58.3)	77.9 (74.1 – 81.4)
**4**	88.3 (83.9 – 92.2)	34.8 (26.3 – 43.2)	25.0 (0.0 – 50.0)	88.9 (86.0 – 91.7)
**5**	83.5 (78.7 – 88.3)	49.2 (40.7 – 58.5)	25.0 (0.0 – 50.0)	96.2 (94.6 – 97.7)
**6**	74.4 (68.7 – 80.0)*	65.3 (56.8 – 73.7)*	25.0 (0.0 – 50.0)	98.3 (97.1 – 99.4)
**7**	66.5 (60.4 – 72.6)*	74.6 (67.0 – 82.2)*	8.3 (0.0 – 25.0)	99.6 (99.0 – 100.0)
**8**	56.1 (50.0 – 62.2)	83.1 (76.3 – 89.0)	8.3 (0.0 – 25.0)	99.8 (99.4 – 100.0)
**9**	50.4 (44.4 – 57.0)	86.4 (80.5 – 92.4)	8.3 (0.0 – 25.0)	100.0 (100.0 – 100.0)
**10**	44.8 (38.7 – 51.3)	90.7 (85.6 – 95.8)	8.3 (0.0 – 25.0)	100.0 (100.0 – 100.0)
**11**	37.4 (31.3 – 43.5)	94.1 (89.8 – 98.3)	0.0 (0.0 – 0.0)	100.0 (100.0 – 100.0)
**12**	94.9 (90.7 – 98.3)	33.5 (27.8 – 39.6)	0.0 (0.0 – 0.0)	100.0 (100.0 – 100.0)
Sensitivity and specificity are presented in % (with corresponding 95% confidence intervals). *The range of the optimal cut-off value according to the Youden index method (see Table 3). aPTT-A - activated partial thromboplastin time determined using STA–PTTA reagent (Roche Diagnostics). aPTT-LA - LAC-sensitive activated partial thromboplastin time. ^†^aPTT-FS is not recommended for LAC-testing				

### LAC testing in patients with heparin or VKA therapy

In 1390 patients of dataset B (LAC positivity rate: 16.3%), 319 patients presented with heparin therapy (up to 1 IU/mL anti-Xa activity). In these patients, a tendency for lower ROC-AUCs to detect LAC was found in all aPTT-A- and aPTT-LA-derived parameters (see Supplementary table 3). Due to the considerably low sample size of patients with heparin therapy, statistical significance was achieved in none of the ROC-AUC differences with the exception of the aPTT-FS (assessed in all patients, P = 0.002).

In patients with VKA (dataset C, N = 2130), the LAC positivity rate ranged between 11.1% and 16.7% (see Supplementary table 3). While the accuracy of the aPTT-A mixing test was lower in patients with INR > 3.51, this effect was not seen in the aPTT-LA mixing test or their derived parameters. Patients with VKA therapy were statistically compared to those patients without evidence of factor deficiency and anticoagulation therapy (dataset A, see Figure 1 and Table 1). Notably, several aPTT-LA derived parameters showed significantly better results in patients with VKA therapy than in the control group (P_range_ < 0.001 to 0.969).

## Discussion

The majority of data assessing the diagnostic capacity of LAC testing is derived from a well-controlled study environment, which possesses several alterations to the daily clinical routine (*10*). Therefore, we have conducted this retrospective cohort study, to assess the predictive capacity of LAC screening and mixing test parameter for detecting a positive result in confirmation tests within the same blood sample. Reassessment of a positive confirmation test (as a criterion for diagnosing APL-syndrome) was not goal of this study.

In patients without factor deficiency and without anticoagulation therapy (dataset A, N = 2312), the LAC positivity rate was 36%. Within dataset A patients, the highest ROC-AUCs for detecting LAC were found by using the aPTT-LA_screen_ and the aPTT-LA mixing test derived parameters. The aPTT-LA ∆Mix–PNP performed significantly better than the dRVVT ∆Mix–PNP. Although guidelines describe the dRVVT as being more specific for detecting LAC or particularly emphasize its usage, a better performance of aPTT-derived testing systems than dRVVT-based parameters can also be found in the literature, with fewer false negative and false positive results or more true positive results (*4*, *6*, *11*, *12*). However, a considerable variability exists between various reagent and analyser combinations, limiting the generalizability of study results (*13*). In 2010, LAC testing data from four consecutive testing surveys, conducted by The North American Specialized Coagulation Laboratory Association (NASCOLA), was published using commercially available lyophilized plasma samples. When assessing a low-titre LAC plasma pool, the dRVVT screening test resulted in 7.5% false negative results and the dRVVT mixing test indicated 9.5% false negatives, while the LAC sensitive aPTT screening test presented with 3.0% false-negative results and the aPTT mixing test resulted in 4.7% false negatives. Further, when assessing a normal plasma pool, the LAC-sensitive aPTT and dRVVT resulted in a similar range of false positive results (7.4% *vs*. 7.8%, respectively) (*11*).

The ROC-AUC of dRVVT_screen_ was higher than those ROC-AUCs of dRVVT-derived mixing parameters. This effect was not observed in aPTT-LA derived parameters. Although these differences were not statistically significant, the use of the dRVVT mixing test should be carefully evaluated. A clear benefit of the dRVVT mixing test was not observed in this cohort. This finding adds to the controversial discussion regarding the clinical need for the mixing test, especially in the context of integrated test systems (*14*–*18*). Generally, mixing tests might increase the specificity while potentially decreasing the sensitivity by diluting effects of specimens with weak antibodies (*19*, *20*). However, when mixing tests are omitted, false negative results related to a very strong LAC, but false positives especially in patients with VKA have also been reported (*21*, *22*). In this regard, the BCSH guideline considers specimens without other causes of prolonged CT, negative mixing tests but positive screening and confirmation tests as LAC positive (*6*). Furthermore, the lupus cofactor has been described as a heterogeneous and imprecisely characterized phenomenon, which is detectable when adding PNP to the PP (*23*, *24*). Therefore, the mixing test might have a significant value in some selected patients, but implies a low cost-effectiveness in widespread use. In this regard, our findings are concordant with the recommendations of the CLSI guidelines, which advocate the usage of the mixing test only in unclear cases (*2*). In our opinion, a predictive model established with LAC screening coagulation tests using linear or non-linear methods might have a better diagnostic accuracy than mixing test studies in a widespread application.

Further, the usefulness of the ICA is controversially discussed in literature (*25*). While the ISTH guideline suggests its usage as an alternative approach, in our evaluation, no benefit has been found for calculating the ICA or the ∆PP–PNP compared to ∆Mix–PNP. This is in line with several recent studies using different patient settings (*12*, *16*, *26*).

Although usage of the aPTT-FS for the mixing test is not recommended in current guidelines, we performed this procedure in 491 patients (including 12 patients with evidence of LAC, dataset A) whose difference to the reference value was the highest in the aPTT-FS among the LAC screening parameters. Typically this occurred in patients with factor deficiency. These patients were presented in the results section, since their deletion would have altered the pre-test probability of a positive confirmation test, and since it is important to make clear the inappropriateness of such an algorithm. Even with an optimal threshold of only 1.75 s, the aPTT-FS ∆Mix–PNP presented with only 66.7% sensitivity, which clearly restricts its usefulness for LAC testing. To prevent a potential impact of the inappropriate aPTT-Actin FS on the overall study results, the diagnostic accuracy analysis of screening tests were split and statistical comparison of aPTT-Actin FS to LAC-sensitive screening tests was avoided.

Interestingly, the FVIII, FIX, FXI, FXII activities and fibrinogen concentrations had some predictive capacity for identifying patients with LAC. For all five parameters, patients with LAC had significantly higher levels than patients without evidence of LAC. This might be based on an acute phase reaction or an inflammatory response reaction, which might be accompanied with the expression of LAC or be based on an infectious genesis of the expression of LAC (*27*). Further, since a relatively large cohort was analysed, small differences between the LAC positive and LAC negative patients yield into statistical significance. Moreover, statistical significance does not necessarily imply clinical relevance and ROC-AUC analysis results are in our case better suited to estimate the effect sizes of LAC testing parameters. In patients on heparin (anti-Xa activity ≤ 1 IU/mL), the diagnostic performance of LAC parameters was remarkably lower than in those without heparin. However, apart from the aPTT-FS, these differences were not statistically significant, which might be related to the relatively small sample size in some subgroups.

Generally, recent guidelines recommend LAC testing after discontinuation of VKA therapy. However, in daily clinical practice LAC testing is also requested in patients under VKA therapy especially as discontinuation of the VKA therapy is not always feasible. Therefore, patients with heparin or VKA were evaluated separately in our statistical analysis. The effect of non-vitamin K antagonist oral anticoagulants (NOACs) was not evaluated, since NOACs were hardly used in Austria during the study period. According to the manufacturer´s instruction sheet (StaClot-LA direction for use, Version 4) the test is insensitive to heparin up to 1 IU/mL due to a heparin inhibitor and no false positive result was found in patients under VKA therapy. In the case of dRVVT confirmation test, no precise information is given in the manufacturer´s instruction sheet on testing patients with heparin or with VKA (#140 0312A). According to our data, LAC testing in patients taking VKA was not statistically impaired in comparison to those patients without VKA (dataset A).

The aPTT-LA-derived parameters presented with partly better ROC-AUCs in patients with VKA than in the control cohort. This finding might indicate that the presence of LAC is more easily unmasked in patients on VKA than in patients without factor deficiency. However, the pre-test probability for having LAC or the positivity rate of confirmation tests might be altered in these selected patients. Further prospective studies are needed to assess the possibility to evaluated LAC testing in patients with VKA. Alternatively, an additional dilution step of patient’s plasma and PNP in patients with VKA might be considered. However, our results are concordant with the literature, which indicates the possibility of LAC testing in patients with VKA (*28*–*30*). Moreover, the usage of proper dRVVT cut-off values or a combination of dRVVT and aPTT-based results for LAC testing in patients with VKA are proposed (*30*).

For this retrospective cohort study approach, several limitations have to be addressed. Although the overall sample size is considerably large, a bigger sample size would be needed for some subgroup analyses. LAC positivity was defined as a positive result of the aPTT-LA or/and dRVVT-based confirmation test in the same plasma sample, thus false positive or false negative confirmation test results could not be ruled out. Finally, in our laboratory the established reference range of the aPTT-LA_screen_ extends up to 49 seconds, which is longer than in most other coagulation laboratories. Therefore, only patients with a higher probability of having LAC were assessed using the aPTT-LA mixing test, which might alter the test’s diagnostic accuracy. However, the diagnostic accuracy of the aPTT-LA_screen_ was significantly higher when compared to the dRVVTscreen (0.84 *vs*. 0.81, P = 0.037) assessed in all dataset A patients.

In conclusion, the pre-test probability of patients with clinically suspected LAC was 36% in patients without factor deficiency or anticoagulation therapy. The aPTT-LA-derived parameters showed a better diagnostic accuracy for identifying LAC than dRVVT-derived parameters. Usage of the mixing test had no further advantage in the LAC diagnostics and the ICA did not improve the performance of the mixing test. According to the results of this study, no impairment was found in patients with VKA, while patients with heparin presented with considerably lower diagnostic accuracy.

## Supplementary material

Supplementary figure 1. The standard operation procedure for LAC testing in our laboratory.

Supplementary table 1. Overview of methods used for assessment of LAC

Supplementary table 2. Descriptive information about quality control materials and coefficients of variation

Supplementary table 3. Coagulation parameters in patients with and without detectable anti-Xa activities

Supplementary table 4. Coagulation parameters in patients with INR assessment
