# Supplementary material for: Testing lupus anticoagulants in a real-life scenario - a retrospective cohort study
Source: Biochem Med (Zagreb). 2017 Aug 28;27(3):030705. doi: 10.11613/BM.2017.030705 (PMC5575653; doi:10.11613/BM.2017.030705)
Supplement: Supplementary file 1 — Supplementary figure 1. The standard operation procedure for LAC testing in our laboratory. [file bm-27-3-030705-S1.pdf]

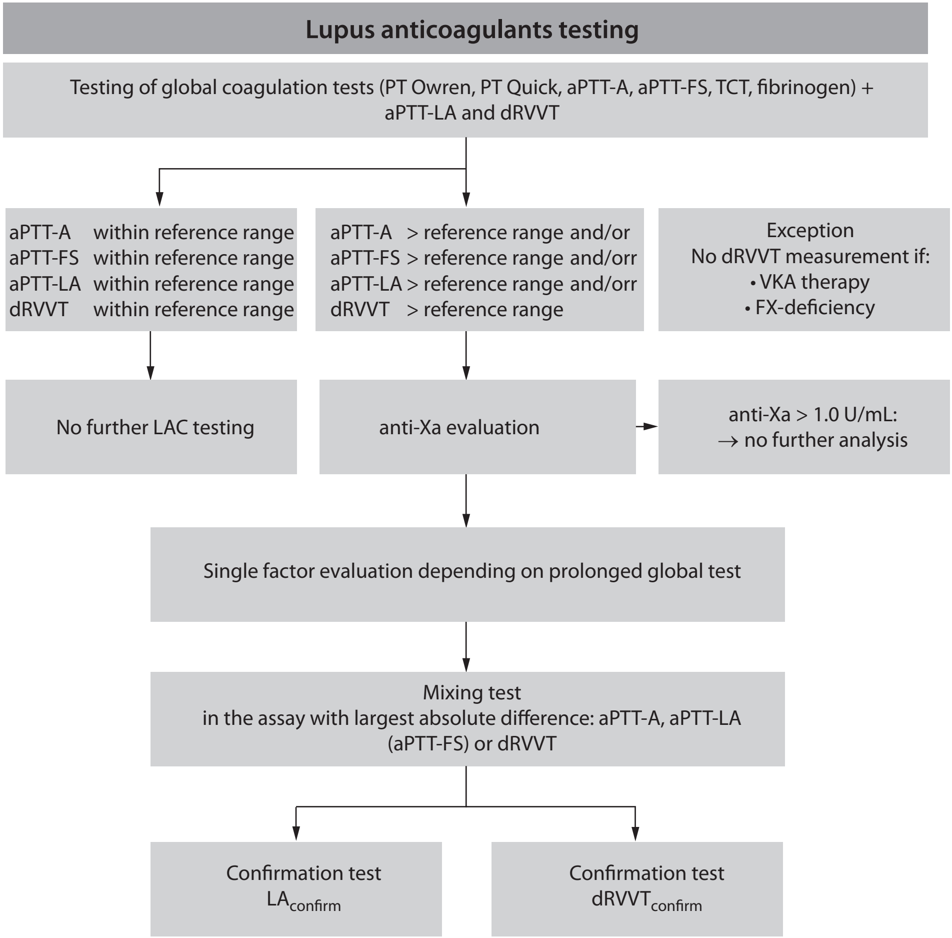

Supplementary figure 1. The standard operation procedure for LAC testing in our laboratory.
PT Owren - prothrombin time according to Owren. PT Quick - prothrombin time according to Quick. aPTT-A - activated partial thromboplastin time determined using STA–PTTA reagent (Roche Diagnostics). aPTT-FS - activated partial thromboplastin time determined using Actin FS (Siemens Healthcare GmbH). TCT - thrombin clotting time. aPTT-LA - LAC-sensitive activated partial thromboplastin time. dRVVT - diluted Russell Viper venom time. VKA - vitamin K antagonists. aPTT-FS is displayed in brackets since this parameter is not recommended for LAC-testing.
