# Supplementary material for: Testing lupus anticoagulants in a real-life scenario - a retrospective cohort study
Source: Biochem Med (Zagreb). 2017 Aug 28;27(3):030705. doi: 10.11613/BM.2017.030705 (PMC5575653; doi:10.11613/BM.2017.030705)
Supplement: Supplementary file 2 — Supplementary table 1. Overview of methods used for assessment of LAC [file bm-27-3-030705-S2.pdf]

SUPPLEMENTARY TABLE 1. Overview of methods used for assessment of LAC

Method	Analyser	Manufacturer	Reagents	Manufacturer	Reference Range
PT Owren	STA-R Evolution*	Diagnostica Stago*	Normotest	Technoclone‡	75 – 140%
PT Quick	STA-R Evolution*	Diagnostica Stago*	Thromborel S	Siemens Healthcare§	80 – 140%
TCT	STA-R Evolution*	Diagnostica Stago*	STA–THROMBIN	Roche Diagnostics‖	< 21 s
aPTT-A	STA-R Evolution*	Diagnostica Stago*	STA–APTT	Roche Diagnostics‖	27 - 41 s
aPTT-LA_{screen}	STA-R Evolution*	Diagnostica Stago*	PTT LA	Roche Diagnostics‖	< 49 s
aPTT-FS	STA-R Evolution*	Diagnostica Stago*	ACTIN FS	Siemens Healthcare§	31 – 38 s
$dRVVT_{screen}$	STA-R Evolution*	Diagnostica Stago*	DRVV SCREEN 2	Life Diagnostics¶	33 – 48 s
$dRVVT_{confirm}$	STA-R Evolution*	Diagnostica Stago*	DRVV CONFIRM	Life Diagnostics¶	< 1.25 ratio**
$LA_{confirm}$	MC10 PLUS†	ABW Medizin und Technik GmbH†	STACLOT-LA	Roche Diagnostics‖	< 3 s††

*Diagnostica Stago S.A.S, Asnières sur Seine, France. †ABW Medizin und Technik GmbH, Lemgo, Germany. ‡Technolclone GmbH, Vienna, Austria. §Siemens Healthcare GmbH, Erlangen, Germany. ‖Roche Diagnostics, Rotkreuz, Switzerland. ¶Life Diagnostics, West Chester, USA. PT Owren - prothrombin time according to Owren. PT Quick - prothrombin time according to Quick. TCT - thrombin clotting time. aPTT-A - activated partial thromboplastin time determined using STA–PTTA reagent (Roche Diagnostics). aPTT-FS - activated partial thromboplastin time determined using Actin FS (Siemens Healthcare GmbH). aPTT-LA - LAC-sensitive activated partial thromboplastin time. dRVVT - diluted Russell Viper venom time. Reference ranges were established by in-house evaluations. **Was considered as being positive when the ratio $dRVVT_{screen}$ to the $dRVVT_{confirm}$ was above 1.25. ††Was considered as being positive when $LA_{confirm}$ decreased CT for more than 3 s.
