# Supplementary material for: Testing lupus anticoagulants in a real-life scenario - a retrospective cohort study
Source: Biochem Med (Zagreb). 2017 Aug 28;27(3):030705. doi: 10.11613/BM.2017.030705 (PMC5575653; doi:10.11613/BM.2017.030705)
Supplement: Supplementary file 3 — Supplementary table 2. Descriptive information about quality control materials and coefficients of variation [file bm-27-3-030705-S3.pdf]

Supplementary table 2. Descriptive information about quality control materials and coefficients of variation

Method	Quality control	Manufacturer	Mean ± SD	CV (%)	Acceptance criteria	Data Source
**PT Owren (%)**	PreciPlus1<br>PreciPlus2	Roche Diagnostics	93.9 ± 5.73<br>46.3 ± 1.9	6.1<br>4.0	81–109<br>37–51	in-house evaluation inter-assay CV (N = 20, measured on 7 days)
**PT Quick (%)**	PreciPlus1<br>PreciPlus2	Roche Diagnostics	87.6 ± 3.2<br>42.4 ± 1.2	3.7<br>2.8	82–110<br>34–48	in-house evaluation inter-assay CV (N = 20, measured on 7 days)
**TCT (s)**	PreciPlus1	Roche Diagnostics	19.1 ± 0.5	2.8	14–20	according to manufacturer´s instruction sheet (V9, 2005-11), N = 21, intra-assay CV
**aPTT-A (s)**	PreciPlus1<br>PreciPlus2	Roche Diagnostics	30.8 ± 0.2<br>52.7 ± 0.5	0.7<br>1.0	28–38<br>48–63	according to manufacturer´s instruction sheet (V11, 2007-12), N = 21, intra-assay CV
**aPTT-LA$_{screen}$ (s)**	PreciPlus1<br>PreciPlus2	Roche Diagnostics	34.2 ± 0.3<br>56.6 ± 1.5	1.0<br>2.6	32.9–36.4<br>50.5–62.8	in-house evaluation inter-assay CV (N = 20, measured on 7 days)
**aPTT-FS (s)**	PreciPlus1<br>PreciPlus2	Roche Diagnostics	32.5 ± 0.6<br>51.1 ± 0.6	1.9<br>1.2	30–37<br>47–53	in-house evaluation inter-assay CV (N = 20, measured on 7 days)
**dRVVT$_{screen}$ (s)**	PreciPlus1<br>PreciPlus2	Roche Diagnostics	47.5 ± 1.2<br>62.0 ± 1.9	2.5<br>3.0	42.4–51.6<br>54.0–65.0	in-house evaluation inter-assay CV (N = 20, measured on 7 days)
**dRVVT$_{confirm}$ (s)**	PreciPlus1<br>PreciPlus2	Roche Diagnostics	45.5 ± 0.6<br>53.7 ± 0.9	1.3<br>1.7	43.2–49.4<br>51.0–59.0	in-house evaluation inter-assay CV (N = 20, measured on 7 days)
**LA$_{confirm}$ (s)**	positive patient samples, normal pool plasma	in-house material	42.5 ± 1.3<br>54.2 ± 2.6	3.0<br>4.7	40–45<br>50–57	in-house evaluation inter-assay CV (N = 20, measured on 7 days)

Roche Diagnostics, Rotkreuz, Switzerland. PT Owren - prothrombin time according to Owren. PT Quick - prothrombin time according to Quick. TCT - thrombin clotting time. aPTT-A - activated partial thromboplastin time determined using STA–PTTA reagent (Roche Diagnostics). aPTT-FS - activated partial thromboplastin time determined using Actin FS (Siemens Healthcare GmbH). aPTT-LA - LAC-sensitive activated partial thromboplastin time. dRVVT - diluted Russell Viper venom time.
