# Supplementary material for: Testing lupus anticoagulants in a real-life scenario - a retrospective cohort study
Source: Biochem Med (Zagreb). 2017 Aug 28;27(3):030705. doi: 10.11613/BM.2017.030705 (PMC5575653; doi:10.11613/BM.2017.030705)
Supplement: Supplementary file 4 — Supplementary table 3. Coagulation parameters in patients with and without detectable anti-Xa activities [file bm-27-3-030705-S4.pdf]

Supplementary table 3. Coagulation parameters in patients with and without detectable anti-Xa activities

	Parameter	Anti-Xa activities < 0.09 IU/mL			Anti-Xa activities ≥ 0.09 IU/mL – 1 IU/mL			P–value[‡]
		**LAC neg N = 870[§]**	**LAC pos N = 20[‖]**	**ROC (95% CI)**	**LAC neg N = 267[¶]**	**LAC pos N = 52[**]**	**ROC[†] (95% CI)**	
**Overall**	**Anti-Xa activities**	0.09 (0.09–0.09)	0.09 (0.09 –0. 09)	np	0.3 (0.2–0.4)	0.2 (0.1–0.4)	np	np
	**aPTT-A**	42.3 (38.4–45.5)	52.9 (45.8–62.1)	0.85 (0.82–0.88)*	42.8 (38.5–47.2)	55.8 (47.5–70.9)	0.86 (0.81–0.90)*	0.673
	**aPTT-FS**	40.6 (38.0 - 43.4)	38.4 (35.8 - 42.4)	0.61 (0.56 - 0.66)*	39.9 (36.2–43.6)	40.4 (36.8–43)	0.54 (0.45–0.62)	0.002
	**aPTT-LA$_{screen}$**	47.0 (42.6–52.3)	64.4 (56.0–77.0)	0.90 (0.87–0.92)*	47.6 (43.0–54.2)	66.8 (56.1–94.2)	0.88 (0.84–0.92)*	0.472
	**dRVVT$_{screen}$**	48.8 (42.3–55.8)	61.0 (53.4–75.4)	0.79 (0.75–0.82)*	52.8 (45.5–60.6)	67.1 (58.6–83.5)	0.79 (0.72–0.86)*	0.991
**Patients grouped together according to the mixing test applied**								
**aPTT-A**		**N = 182**	**N = 31**		**N = 62**	**N = 4**		
	**aPTT-A**	45.6 (44.0–48.6)	56.3 (49.8–61.4)	0.84 (0.76–0.91)*	46.8 (44.3–49.8)	67.0 (44.1–111.0)	0.56 (0.19–0.94)	0.687
	**ΔMix–PNP**	3.5 (1.7–5.0)	10.7 (8.6–15.4)	0.93 (0.87–0.97)*	4.8 (3.2–6.3)	12.3 (8.5–16.0)	0.88 (0.79–0.96)*	0.542
	**ICA**	7.7 (3.7–10.7)	19.4 (15.4–26.1)	0.91 (0.84–0.96)*	10.1 (6.8–13.1)	17.2 (15.1–18.2)	0.82 (0.59–0.97)*	0.559
**aPTT-LA**		**N = 81**	**N = 93**		**N = 27**	**N = 29**		
	**APTT-LA$_{screen}$**	57.5 (54.4–62.5)	70.5 (63.1–82.4)	0.81 (0.74–0.88)*	59.9 (57.0–66.7)	75.6 (65.6–122.3)	0.76 (0.62–0.89)*	0.531
	**ΔMix–PNP**	6.8 (4.5–10.1)	19.4 (10.4–33.2)	0.87 (0.81–0.92)*	8.6 (4.6–12.8)	17.1 (12.6–42.1)	0.83 (0.71–0.93)*	0.551
	**ICA**	12.1 (7.4–16.9)	27.3 (17.8–37.3)	0.85 (0.79–0.90)*	13.4 (6.9–19.0)	23.3 (18.3–38.7)	0.82 (0.70–0.92)*	0.612
**dRVVT**		**N = 217**	**N = 71**		**N = 98**	**N = 18**		
	**dRVVT$_{screen}$**	61.7 (56.4–70.1)	75.9 (64.0–95.3)	0.75 (0.68–0.82)*	62.0 (59.1–71.8)	75.0 (66.9–85.7)	0.76 (0.62–0.88)*	0.618
	**ΔMix–Norm**	6.6 (4.3–11.3)	14.7 (9.8–26.8)	0.79 (0.73–0.84)*	7.4 (5.1–11.2)	13.2 (9.3–17.1)	0.75 (0.62–0.86)*	0.763
	**ICA**	10.5 (7.4–16.6)	18.8 (14.9–31.8)	0.77 (0.71–0.83)*	12.3 (8.3–15.6)	18.0 (12.8–20.6)	0.72 (0.59–0.83)*	0.650

np - not performed. *statistically significantly better than random guessing. ROC - area under the receiver operating characteristics curve, presented with 95% confidence intervals (CI). [‡]assessment of those patients with and without heparin therapy using the Venkatraman test for comparison of unpaired ROC-AUCs, including patients with mixing test performed using the aPTT-FS (data not shown due to low percentage of LAC positives): [§]N = 390, [‖]N = 6, [¶]N = 80, [**]N = 1. aPTT-A - activated partial thromboplastin time determined using STA–PTTA reagent (Roche Diagnostics). aPTT-FS - activated partial thromboplastin time determined using Actin FS (Siemens Healthcare GmbH). aPTT-LA - LAC-sensitive activated partial thromboplastin time. dRVVT - diluted Russell Viper venom time. PNP - pooled normal plasma. ICA - index of circulating anticoagulant.
