# Supplementary material for: Testing lupus anticoagulants in a real-life scenario - a retrospective cohort study
Source: Biochem Med (Zagreb). 2017 Aug 28;27(3):030705. doi: 10.11613/BM.2017.030705 (PMC5575653; doi:10.11613/BM.2017.030705)
Supplement: Supplementary file 5 — Supplementary table 4. Coagulation parameters in patients with INR assessment [file bm-27-3-030705-S5.pdf]

Supplementary table 4. Coagulation parameters in patients with INR assessment

	Parameter	Patients without VKA*			INR 1.3–2.0			INR 2–3.5			INR > 3.5		
		LAC neg	LAC pos	ROC (95% CI) (P)	LAC neg	LAC pos	ROC(95% CI) (P)†	LAC neg	LAC pos	ROC (95% CI) (P)†	LAC neg	LAC pos	ROC† (95% CI) (P)†
**Overall**		**N = 1484‡**	**N = 828‡**		**N = 451§**	**N = 85‖**		**N = 1147¶**	**N = 138****		**N = 225††**	**N = 45‡‡**	
	**INR**				1.6 (1.5–1.8)	1.5 (1.4–1.7)		2.6 (2.3–2.9)	2.7 (2.4–3.1)		4 (3.7–4.6)	4.4 (3.9–5.2)	
	**aPTT-A (s)**	43.5 (41.0–46.2)	48.8 (44.4–56.5)	0.76 (0.74–0.78) (np)	43.9 (39.8–48.1)	56.2 (48.1–69.5)	0.84 (0.80–0.88) (P = 0.008)	45.7 (41.8–51)	64.4 (56.1–80.3)	0.90 (0.88–0.92) (P < 0.001)	55.3 (48.4–65.9)	81.7 (66.5–102.9)	0.82 (0.75–0.88) (P = 0.501)
	**APTT-LA$_{screen}$ (s)**	49.9 (45.8–54.4)	61.2 (54.8–72.3)	0.84 (0.82–0.86) (np)	49.8 (44.8–56)	67.2 (57.3–82)	0.87 (0.83–0.90) (P=0.210)	52.1 (47.3–59)	83.8 (69.1 –110.4)	0.92 (0.90–0.95) (P < 0.001)	64.4 (56.1–77)	94.9 (75.9–128.6)	0.842 (0.78–0.90) (P = 0.314)
	**aPTT-FS (s)**	39.6 (36.7–41.9)	37.0 (34.2–40.3)	0.65 (0.62–0.67) (np)	43.2 (40.0–46.8)	44.5 (40.0–54.0)	0.57 (0.50–0.64) (P < 0.001)	49.5 (45.8–53.9)	52.7 (47.3–58.8)	0.61 (0.56–0.66) (P < 0.001)	59.9 (54.6–67.7)	62.5 (58.3–73)	0.60 (0.52–0.69) (P < 0.001)
**Patients grouped together according to the mixing test applied:**													
**aPTT–A**		**N = 512**	**N = 110**		**N = 121**	**N = 25**		**N = 165**	**N = 17**		**N = 34**	**N = 10**	
	**aPTT-A (s)**	45.0 (43.4–47.6)	50.8 (47.0–57.4)	0.78 (0.73–0.83) (np)	47 (44.5–51)	56.2 (49.4–70.4)	0.78 (0.67–0.88) (P = 0.997)	48.9 (45.6–55.6)	66.2 (56.6–86.1)	0.84 (0.74–0.92) (P = 0.780)	66.7 (55.4–78.7)	78 (67.8–141.6)	0.72 (0.56–0.87) (P = 0.300)
	**ΔMix–PNP (s)**	4.3 (2.6–6.2)	8.2 (5.5–11.1)	0.79 (0.75–0.84) (np)	4.7 (2.8–6.6)	9.6 (6.3–16.6)	0.81 (0.70–0.90) (P = 0.892)	3.5 (1.6–5.5)	12.8 (7.3–19.8)	0.83 (0.69–0.95) (P = 0.519)	4.8 (1–9.6)	8.3 (6.3–12.6)	0.69 (0.52–0.85) (P = 0.216)
	**ICA (%)**	9.3 (5.8–12.9)	16.1 (11.3–20.8)	0.77 (0.72–0.82) (np)	9.5 (6–13.3)	15 (11.5–20.3)	0.76 (0.66–0.86) (P = 0.960)	7.1 (2.8–10.5)	17.3 (10.8–28.8)	0.82 (0.69–0.95) (P = 0.478)	7.3 (2–11.9)	9.4 (8.6–14.8)	0.63 (0.44–0.81) (P = 0.163)
**aPTT–LA**		**N = 375**	**N = 476**		**N = 94**	**N = 53**		**N = 241**	**N = 114**		**N = 68**	**N = 30**	
	**APTT-LA$_{screen}$ (s)**	56.4 (53.4–60.3)	67.5 (59.3–81)	0.80 (0.77–0.83) (np)	58.6 (54.7–64.1)	70.9 (59–89.8)	0.73 (0.65–0.81) (P = 0.126)	63.4 (58.8–72.3)	89.1 (72.4–116.6)	0.83 (0.78–0.87) (P = 0.537)	74.4 (63.4–85.8)	114 (91.1–130.4)	0.86 (0.77–0.93) (P = 0.015)
	**ΔMix–PNP (s)**	6.7 (4.7–9.6)	15.8 (10.3–25.3)	0.84 (0.82–0.87) (np)	6.3 (3.4–9.7)	14.1 (8.6–22.4)	0.79 (0.71–0.86) (P = 0.969)	4.9 (2.6–8.8)	29.2 (11.8–46)	0.90 (0.87–0.94) (P < 0.001)	6 (2.7–9.3)	25.8 (20.9–39.8)	0.94 (0.87–0.98) (P = 0.010)
	**ICA (%)**	11.9 (8.3–16.2)	23.2 (16.4–33.6)	0.83 (0.80–0.85) (np)	11.1 (6.2–15.5)	20.4 (15.2–29.4)	0.78 (0.70–0.86) (P = 0.331)	7.9 (4.2–12.9)	30.1 (16.2–44.7)	0.90 (0.86–0.93) (P = 0.008)	7.8 (3.4–11.9)	27 (18.9–33.7)	0.89 (0.81–0.95) (P = 0.252)

np - not performed. *patients without evidence of factor deficiency and anticoagulation therapy (dataset A, see Figure 1/ Table 1). ROC - area under the receiver operating characteristics curve, presented with 95% confidence intervals (CI). †comparison with patients without VKA using the Venkatraman test for unpaired ROC-AUCs. ‡including patients with the mixing test using the aPTT-FS or dRVVT$_{screen}$ (data shown in Table 1); including patients with the mixing test performed using the aPTT-FS (data not shown due to low percentage of LAC positives): §N = 236, ‖N = 7, ¶N = 741, **N = 7, ††N = 123, ‡‡N = 5. aPTT-A - activated partial thromboplastin time determined using STA–PTTA reagent (Roche Diagnostics. aPTT-LA - LAC-sensitive activated partial thromboplastin time. dRVVT - diluted Russell Viper venom time. PNP - pooled normal plasma. ICA - index of circulating anticoagulant.
